# Bioprinting-By-Design of Hydrogel-Based Biomaterials for In Situ Skin Tissue Engineering

**DOI:** 10.3390/gels11020110

**Published:** 2025-02-03

**Authors:** Alisa Douglas, Yufei Chen, Margarita Elloso, Adam Levschuk, Marc G. Jeschke

**Affiliations:** 1 Department of School of Biomedical Engineering, McMaster University, Hamilton, ON L8S 4L8, Canada; alisa.douglas@taari.ca; 2 David Braley Research Institute, Hamilton, ON L8L 2X2, Canada; yufei.chen@taari.ca; 3 Hamilton Health Sciences, Hamilton, ON L8L 0A4, Canada; margarita.elloso@taari.ca; 4 Department of Surgery, McMaster University, Hamilton, ON L8S 4L8, Canada; 5 Schulich School of Medicine and Dentistry, Western University, London, ON N6A 5C1, Canada; alevschuk2025@meds.uwo.ca

**Keywords:** hydrogel, bioprinting, in situ, burns, skin regeneration, bioink, tissue engineering, biomaterials

## Abstract

Burns are one of the most common trauma injuries worldwide and have detrimental effects on the entire body. However, the current standard of care is autologous split thickness skin grafts (STSGs), which induces additional injuries to the patient. Therefore, the development of alternative treatments to replace traditional STSGs is critical, and bioprinting could be the future of burn care. Specifically, in situ bioprinting offers several advantages in clinical applications compared to conventional in vitro bioprinting, primarily due to its ability to deposit bioink directly onto the wound. This review provides an in-depth discussion of the aspects involved in in situ bioprinting for skin regeneration, including crosslinking mechanisms, properties of natural and synthetic hydrogel-based bioinks, various in situ bioprinting methods, and the clinical translation of in situ bioprinting. The current limitations of in situ bioprinting is the ideal combination of bioink and printing mechanism to allow multi-material dispensing or to produce well-orchestrated constructs in a timely manner in clinical settings. However, extensive ongoing research is focused on addressing these challenges, and they do not diminish the significant potential of in situ bioprinting for skin regeneration.

## 1. Introduction

Patients with wounds, trauma, burns, and even cancer often require coverage of dermal and epidermal defects [1]. For decades, the gold standard in plastic reconstruction, and particularly for burn patients, has been the use of split thickness skin grafts (STSGs) [1,2]. Burn injuries are among the most common trauma injuries worldwide, with a detrimental impact on the entire body. According to the World Health Organization, approximately 9 million burn injuries occur annually, and 180,000 are fatal [3]. Furthermore, up to 2.3 million cases globally have been reported to have large body surface area burns requiring surgical interventions including excisions and skin grafts [4]. STSGs require a healthy skin harvest for wound coverage, which presents significant challenges as this induces additional injuries to the patient, causing pain and scarring. A major disadvantage of this approach is the limited availability of donor sites to cover large burn injuries or trauma-associated wounds. Hence, it is essential to develop novel approaches to replace skin grafting to mitigate pain, scarring, and mortality risks.

In this context, tissue engineering and in situ bioprinting offer promising techniques for the fabrication of skin substitutes, providing rapid wound coverage and promoting tissue regeneration, both of which are critical for patient survival [2,5]. In situ bioprinting represents a cutting-edge technology capable of producing patient-specific skin substitutes. This technique involves printing directly on the wound, making it suitable for surgeons to use in the operating room in real time. Bioprinters contain a bioink and a crosslinking mechanism for polymerization [6]. The bioink typically consists of therapeutic materials, with hydrogel-based inks composed of synthetic polymeric materials or natural proteins being the most common. Various materials require different crosslinking mechanisms, each presenting its own advantages and disadvantages. Additionally, cells and other therapeutic agents can be encapsulated in bioinks to enhance wound healing capabilities [7]. The primary types of bioprinting mechanisms include extrusion, inkjet, and laser assisted bioprinting (LAB), which can be delivered through various modalities such as handheld, robotic, and minimally invasive printers. Despite this being a well-studied area that has garnered increasing attention, there are challenges related to clinical translation that must be addressed before this technology can be implemented in the medical field.

**Figure 1:**
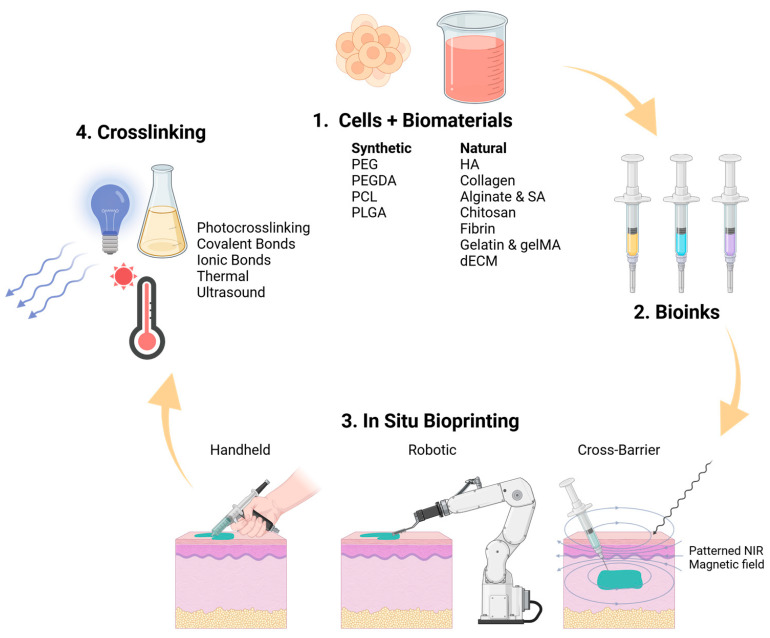
Overview of the processes involved in in situ bioprinting for skin regeneration: biomaterials, formulation of bioinks, types of in situ bioprinting, and crosslinking mechanisms. Created in BioRender. Jeschke, M. (2025) https://BioRender.com/p78e422 (accessed on 20 January 2025).

In this review, we intend to provide an in-depth summary of the recent advancements in the field of skin regeneration during the past six years with the use of in situ bioprinting. The review discusses the critical aspects which must be considered for in situ bioprinting to regenerate skin, including the types of crosslinking mechanisms, properties of various synthetic and natural biomaterials, and the different categories of in situ bioprinting (Figure 1).

## 2. Crosslinking Mechanisms

Crosslinking mechanisms are crucial to consider when choosing a bioprinting method. Crosslinking is required to polymerize the printed construct typically through physicochemical reactions and can be achieved through various forms, including light, ionic bonding, covalent bonding, temperature, pH, and ultrasound. Each method has its own advantages and drawbacks, with trade-offs between cytocompatibility, crosslinking speed, and mechanical strength. Certain materials can only be crosslinked using specific mechanisms, therefore it is essential to determine the ideal crosslinking method based on the required material and desired properties.

### 2.1. Light

Photocrosslinking is one of the most used methods due to its fast-crosslinking speed and higher efficiency. Materials must be intrinsically photo-reactive or chemically modified to be photoreactive [6]. Commonly used photocrosslinkable biomaterials include gelatin methacrylolyl (GelMA), polyethylene glycol diacrylate (PEGDA), galactoglucomannan methacrylates, methacrylated hyaluronic acid (HA), and methacrylated chitosan [6,8]. GelMA and PEGDA are the most used photo-reactive materials, which can be crosslinked in presence of a water-soluble photoinitiator and ultraviolet (UV), blue, or visible light [6,9].

Photoinitiators are required to initiate the reaction as they induce a free radical chain process, causing the prepolymers to crosslink once exposed to light [10]. There are two types of phoinitiators. Type I can only be used with UV light and cleave once exposed. On the other hand, type II can be used with a greater range of wavelengths, allowing them to be used with UV and visible light. Type II photoinitiators are commonly used for higher cytocompatibility as visible light is less toxic, resulting in higher cell viability [6]. Examples of type II photoinitiators includes lithium phenyl (2,4,6-trimethylbenzoyl) phosphinate (LAP), eosin Y, and camphorquinone. LAP is one of the most used photoinitiators as it allows for visible light crosslinking, thereby reducing toxicity. Irgacure 2959 is the most widely used type I photoinitiator, however studies have shown that LAP resulted in higher cell viability than Irgacure 2959 when both were used with UV light. Additionally, another study found that LAP maintains superior cytocompatibility over time at higher concentrations compared to Irgacure 2959 [11].

Depending on the photoinitiator chosen, different light sources and wavelengths can be used, including visible light (wavelengths: 380–700 nm), UV (365 nm and 405 nm are most common), and near infrared (NIR) (wavelengths: 760–1400 nm) [12]. In addition, various parameters can be adjusted to alter polymerization speed such as the light source, light intensity, and exposure time [6].

### 2.2. Ionic Bonds

Ionic crosslinking occurs when polymers with opposite charges form an ionic bond. This is a common method due to its speed and because it does not require extreme conditions, making it achievable at room temperature and neutral pH [6]. However, this method usually results in lower mechanical strength and poor stacking stability as ionic bonds are not as strong [6]. Alginate and sodium alginate are widely used materials which undergo ionic crosslinking through calcium salts, such as calcium chloride, calcium carbonate, or calcium sulphate [6]. Calcium is commonly used as it is highly water soluble and results in faster polymerization [6]. Other ions used for crosslinking sodium alginate include barium, zinc, ferric, and strontium [13,14]. Although ionic crosslinking is frequently used and crosslinks quickly, the concentration of ions can affect cell viability, as high calcium concentrations are toxic and can damage cell membranes [15].

### 2.3. Covalent Bonds

The formation of covalent bonds is stronger than ionic bonds, thereby resulting in greater mechanical strength compared to ionic crosslinking [16]. However, this process is also slower. Common materials which undergo covalent crosslinking include Poly(ethylene glycol) (PEG), Polycaprolactone (PCL), hyaluronic acid, collagen, and fibrin, requiring chemical crosslinking agents such as glutaraldehyde (GA), genipin, and thrombin [17,18,19,20,21,22,23].

### 2.4. Thermal

Thermosensitive materials can crosslink through a temperature change. This method is advantageous for biological applications such as drug delivery, injections, or implantations as numerous materials crosslink at body temperature (37 °C) [24]. However, the shortcomings include slower gelation time, less precise degree of crosslinking, and heat can negatively affect cell viability [6]. Furthermore, unlike covalent and ionic bonds, thermal crosslinking is a form of physical crosslinking, which is a reversible process. This means that most biomaterials will transition to a gel at 37 °C, however, it can revert back into a liquid once the temperature cools down. Examples of thermally crosslinked materials include decellularized skin, agarose, gelatin, collagen, chitosan, poloxamers, N-isopropylacrylamide based co-polymers, hyaluronic acid, and poly (ε-caprolactone) poly (L-lactide) diol [25,26,27,28,29].

### 2.5. pH

pH is another method for crosslinking, although it is not as common. Adjusting the pH can form ionic crosslinking, causing the polymers to bond through electrostatic interactions as varying the pH results in a change in the isoelectric point [6]. Acidic solutions can result in electrostatic crosslinking, while basic solutions result in a hydrogen bond-based crystalline network [30]. Chitosan is a commonly used material which crosslinks based on pH change.

### 2.6. Ultrasound

The use of ultrasound (US) for crosslinking is extremely rare, although it is possible. Similar to photocrosslinking, US can crosslink a bioink blended with an initiator [31]. US causes thermal dissociation of initiator, which results in crosslinking [31]. It is also feasible to crosslink a polymer without an initiator, however, this is uncommon [31]. F. Zhou et al. used both US and UV light sequentially to crosslink GelMA hydrogel which contains copper (Cu)-containing bioactive glass nanoparticles (Cu-BGn) [32].

**Figure 2:**
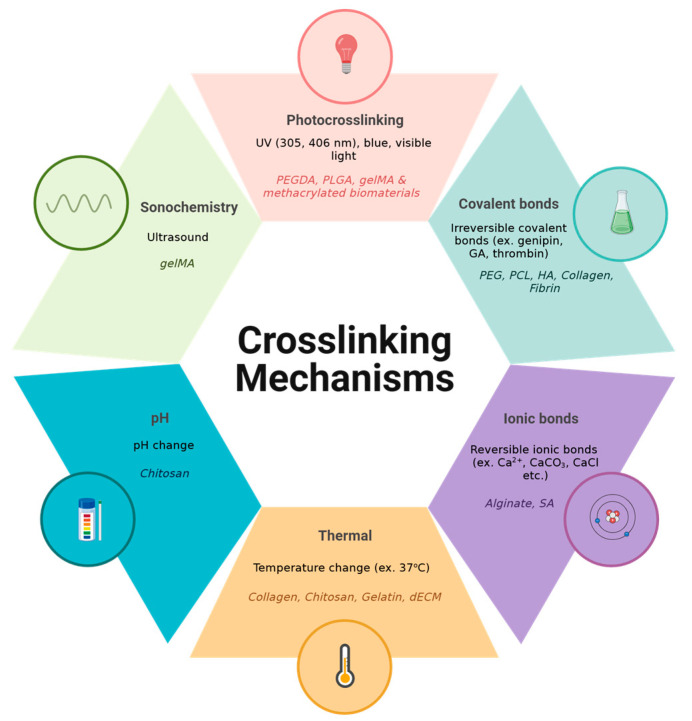
Summary of various crosslinking mechanisms and the biomaterials associated with each category. Created in BioRender. Jeschke, M. (2025) https://BioRender.com/t36a954 (accessed on 10 January 2025).

As discussed above, various crosslinking mechanisms can be used depending on the application and the biomaterials used (Figure 2). For in situ bioprinting, covalent and ionic crosslinking are the most common methods, as chemical crosslinkers can be mixed with the bioink immediately and begin to crosslink once deposited on the wound. This is typically conducted by storing the bioink and crosslinker separately, which are mixed once extruded. This crosslinking mechanism is not only the easiest and most convenient for in situ bioprinting, but is also applicable to widely used biomaterials such as sodium alginate, fibrin, collagen, and hyaluronic acid. Photocrosslinking is also a possibility, however, prolonged exposure to UV light on the wound and skin can be harmful. Thermal crosslinking is ideal for injectable hydrogels, as they can be injected as a liquid and crosslink at body temperature. However, for in situ bioprinting to promote wound healing, the bioink is deposited on the skin, which is not as warm as it is exposed to room temperature. Therefore, thermal crosslinking would take significantly longer in this context and is therefore less commonly used. pH crosslinking is also not applicable for this application as the acid and bases involved would cause more harm to the wound. Lastly, the use of US is extremely rare, it would be difficult to use in a clinical setting, and it is not applicable to most biomaterials. Although each crosslinking mechanism has its own advantages and disadvantages, they must be considered for each application, as not all mechanisms are suitable for in situ bioprinting.

## 3. Hydrogel-Based Biomaterials and Their Wound Healing Mechanisms

The biomaterials used are a crucial component of bioprinting. To be classified as a bioink, it is mandatory for the biomaterials to contain cells, otherwise they are considered biomaterial inks [33,34]. Numerous biomaterials are considered ideal candidates for use as hydrogel-based inks. Hydrogels are composed of a 3D network structure of hydrophilic polymers, allowing them to mimic the extracellular matrix structure and retain a high-water content. These characteristics make hydrogels well-suited for encapsulating cells and allow them to be bioprinted in various structures. Given their unique properties, hydrogel-based bioinks and hydrogel-based biomaterials have become an attractive option for bioprinting in tissue engineering applications.

**Figure 3:**
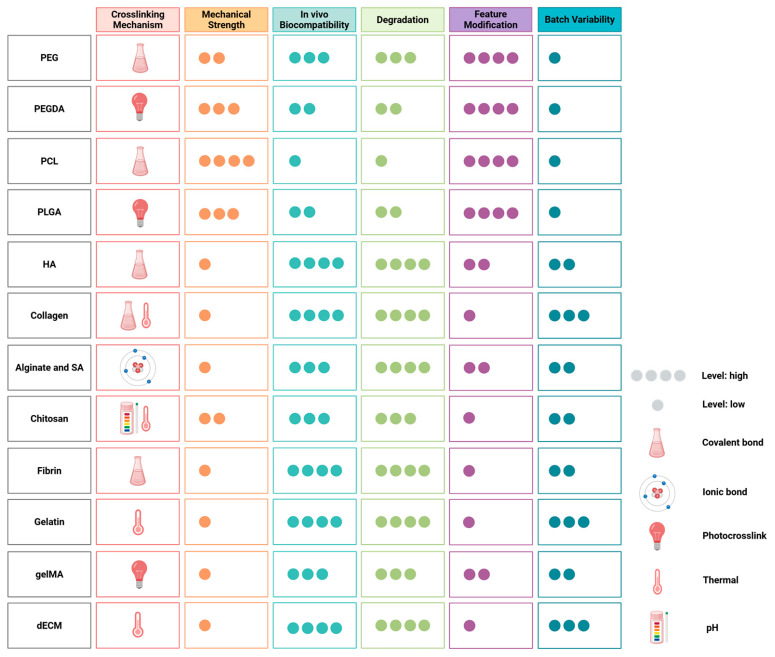
A summary of the various properties of biomaterials, including crosslinking mechanisms, mechanical strength, in vivo biocompatibility, degradation rate, modifiability, and batch variability. The dots represent varying levels of strength, with four dots indicating the highest level and one dot indicating the lowest level. Created in BioRender. Jeschke, M. (2025) https://BioRender.com/m71q248 (accessed on 7 January 2025).

Based on their origin and chemical nature, biomaterials can be further grouped into two categories: synthetic and natural. Synthetic biomaterials use polymers with desired properties to create scaffolds. Key properties to be considered for in situ bioprinting include the degradation rate, biocompatibility, crosslinking rate, viscosity, hydrophobicity, mechanical strength, printing speed, and fabrication technique. Additionally, many bioinks are composites of multiple polymers in blocks, named as co-polymers. A significant advantage of synthetic polymers is the ability to easily tune its mechanical properties through chemical modifications of the available moieties and/or introduction of other polymers to form co-polymers enabling additional target functionalities. Moreover, reproducibility between batches is consistent, which is advantageous for large-scale manufacturing. In contrast, natural materials consist of the crucial proteins abundantly found within the native skin, supplementing the tissue with desired biological factors to promote the integration of the printed constructs and eventually enhance skin regeneration. However, natural materials often vary between batches due to the source, rendering some challenges for larger-scale production. In conclusion, both types of materials have valuable properties and drawbacks that must be considered when developing the hydrogel-based bioink, which are summarized in Figure 3.

### 3.1. Synthetic

#### 3.1.1. PEG

Poly(ethylene glycol), also known as PEG, is a frequently used material in biofabrication. PEG has an extremely broad range of molecular weights, from a couple of kilodaltons, with each repeating unit approximately 44 kDa, to high molecular weights in the range of 2000–20,000 kDa [35]. PEG can be manufactured in various structures including linear, multi-arm, and branched, although the traditional form is linear [36]. Although PEG is known for its hydrophilicity, it is technically amphiphilic and therefore also contains hydrophobic properties [35,37]. This versatility results in a wide range of applications as it can be dissolved in numerous organic solvents as well as water [35]. Its molecular weight also affects its rheological behaviour: at lower molecular weights (~200 kDa), PEG behaves as a Newtonian fluid, while at higher molecular weights, it exhibits shear-thinning behaviour [38]. This shear-thinning behaviour is vital for bioprinting, as the printing process exerts shear stress on the ink, requiring its viscosity to decrease in order to successfully extrude the bioink. After extrusion, polymerization of PEG is commonly performed through covalent crosslinking, such as genipin [19].

PEG is widely used due to its biocompatibility, highly hydrophilic features, and it is non-fouling, thus aiding in reducing protein adsorption [39]. Due to its amphiphilic nature, it is commonly used to encapsulate cells and coat materials to lower cell adhesion and protein adsorption. PEG is also FDA approved, therefore it is ideal for clinical applications.

#### 3.1.2. PEGDA

Poly(ethylene glycol) diacrylate (PEGDA), another common biomaterial, is synthesized through the acrylation of PEG [40,41]. PEGDA has an extremely slow degradation rate, consequently it is commonly used in applications where long-term degradation is desired, or as a short-term, temporary, and nondegradable material which would be removed after experimentation [41]. PEGDA exhibits higher mechanical strength, making it ideal for applications requiring materials to withstand greater loads [41]. PEGDA contains molecular weights similar to PEG, encompassing a wide range up to 20,000 kDa [35]. Its structure is also similar to that of PEG, as it can be linear or branched, though it is commonly found as a linear chain and is hydrophilic. PEGDA has been shown to be a shear-thinning fluid, which is essential for bioprinting [42]. Furthermore, PEGDA contains reactive acrylate groups utilized for photocrosslinking under UV light [41]. Similar to PEG, PEGDA has numerous desirable properties: molecular weights over 400 Da are non-toxic, biocompatible, and approved by the FDA, making this beneficial for clinical applications [41]. Y. Tianyuan et al. developed a handheld bioprinter for in situ printing on wounds. The device contains three nozzles for spraying, extrusion-based printing, and electrospinning. The first nozzle sprays PEGDA to quickly cover the wound and create a flat surface, followed by extrusion of a gelatin and sodium alginate blend through the second nozzle, and electrospinning of PCL through the third nozzle. Finally, UV light at 365 nm was used for rapid crosslinking [43].

#### 3.1.3. PCL

Polycaprolactone (PCL) is a highly crystalline, hydrophobic, and aliphatic material [44,45]. PCL is a linear polymer with molecular weights ranging from 530 to 630,000 g/mol. Due to its high hydrophobicity, it can dissolve in toluene, benzene, chloroform, dichloromethane, and carbon tetrachloride at room temperature, but is insoluble in water and alcohols [45]. PCL has an intrinsic viscosity of 0.9 cm^3^/g and is shear-thinning when exposed to higher shear stress [46]. Due to PCL’s high crystallinity and mechanical strength, it is slightly less biocompatible and has a slower degradation rate. PCL is biodegradable but the process is significantly slower than polylactides as it contains less ester bonds, therefore degrading slower through enzymes and hydrolysis, which can ultimately take months to several years [45]. PCL exhibits semicrystalline properties at room and body temperatures, hence it is tailored for applications involving stiffer tissues or for temporary use for support and mechanical strength. Furthermore, PCL is commonly polymerized via chemical crosslinking, including genipin and peroxide [20,21]. A. Mostafavi et al. printed a bioink blend consisting of PCL, hydroxyapatite (HAp), and zinc oxide (ZnO) for bone remodeling in mice. ZnO provides the scaffold with antibacterial properties, while PCL and HAp provide mechanical strength and structure [47].

#### 3.1.4. PLGA

Poly(lactic-co-glycolic acid), also known as PLGA, is a copolymer consisting of lactic acid and glycolic acid, which is synthesized through ring-opening polymerization [39]. Their molecular weight also varies significantly, ranging from 10 kDa up to 100 kDa [48]. PLGA is a linear chain, and its hydrophilicity is determined by the ratio between the two acids, as a higher concentration of lactic acid compared to glycolic acid results in lower hydrophilicity [48]. Furthermore, the rheological behaviour of PLGA is dependent on its concentration: at lower concentrations, PLGA behaves as a Newtonian fluid, and at higher concentrations (approximately 35–40%) it is shear-thinning [49]. PLGA is a commonly used biomaterial as it is FDA approved, biocompatible, and biodegradable, however, its degradation rate is slow, making PLGA more suited for long term applications [50]. Similar to its hydrophilicity, PLGA’s mechanical strength can also be adjusted by altering the ratio of lactic acid to glycolic acid concentration, with higher concentration of lactic acid resulting in greater mechanical strength and stiffness [39]. The ease of altering the ratio of lactic acid to glycolic acid results in PLGA having a wide range of molecular weights and physical properties [50]. E. Pagan et al. used stereolithography (SLA) printing, microfluidics, and hydraulic extrusion in a handheld bioprinter to extrude various materials to test the device’s printability, including PEGDA-laponite, PLGA, alginate, PNIPAM, and GelMA. UV light at 405 nm was used for crosslinking most bioinks and calcium chloride was used for alginate [51].

### 3.2. Natural

#### 3.2.1. Hyaluronic Acid

Hyaluronic acid (HA) is a natural polyanionic polymer in the body, commonly found in joints, eyes, heart valves, as well as skin [52]. HA is a linear polysaccharide, and its main advantage lies in its ability to trap large amounts of water molecules, aiding in hydration and lubrication [52,53]. The molecular weight of HA varies largely depending on the source, however in most animal tissues it can reach up to 20,000 kDa [53]. Although, it was found that HA with molecular weights between 20 and 200 kDa are usually involved in wound healing [53]. HA is highly hydrophilic, aiding in its ability to retain large amounts of water. It is shear-thinning, has viscoelastic behaviour, and its viscosity increases based on its molecular weight and concentration. As HA is naturally found within the skin’s extracellular matrix (ECM), it is highly biocompatible and is favourable to use in bioinks. It aids in biological processes such as wound healing, cell signaling, and matrix organization. CD44 is a cell-surface glycoprotein that binds to hyaluronan, its principal ligand, which activates the receptor to promote cell proliferation, migration, and adhesion. CD44 is also involved in the cellular internalization of HA degradation products [54]. A study has shown that blocking CD44 on the surface of macrophages resulted in a delayed healing process in lung inflammation. Furthermore, the binding of hyaluronan to CD44 recruits fibroblast migration and proliferation from surrounding tissues to the wound site [55]. Another main receptor, the Receptor for Hyaluronan-Mediated Motility (RHAMM), also known as CD168, has been proven to activate fibroblast proliferation in vitro [56]. Depending on its molecular weight, HA can have different effects. Lower molecular weight HA has been shown to be pro-inflammatory, while higher molecular weight HA has anti-inflammatory properties [57].

JY. Zhao et al. investigated the wound healing properties of HA in pigs and found that HA aided in tissue regeneration as the results were superior compared to autograft and porcine acellular dermal matrix. In addition, it was observed that the HA treatment resulted in greater elasticity and collagen content in the wound compared to other materials [58]. HA can be modified through their carboxyl and hydroxyl groups, however its mechanical strength is insufficient for standalone use. Therefore, it must be blended with other materials to provide structure and support [58,59]. Furthermore, HA is commonly crosslinked via covalent bonds using GA, however, divinyl sulfone (DVS) has also been used [22,23]. N. Hakimi et al. developed a multi-material portable printer for depositing small sheets in situ. The printer was used to extrude an ink consisting of alginate, fibrin, collagen, and HA on full thickness wounds in murine and porcine models. Finally, calcium chloride and thrombin were used for crosslinking [60]. In another study, G. Shin et al. used a syringe to deposit an HA-resveratrol blend to inhibit and reduce tumour growth in mice. The syringe contains HA-tetrazine and HA-trans-cyclooctene, which are stored in separate compartments. During deposition, the two materials mix and rapidly crosslink [61].

#### 3.2.2. Collagen

Collagen is commonly found in connective tissues and is the most abundant protein in animals, particularly in human skin [62]. Numerous types of collagen have been identified, however type I is the most abundant in the body and skin, providing structure, strength, and elasticity [63]. Type I collagen has molecular weights between 250 and 300 kDa and has a unique structure consisting of polypeptides arranged in bundles, creating a triple helix [62,64]. Similar to HA, collagen is shear-thinning, hydrophilic, and typically crosslinked with GA, however, its thermosensitivity also allows for thermocrosslinking at 37 °C [16,17,65]. Collagen has been widely used in bioinks due to its abundance in the skin ECM and its involvement in many physiological interactions, including cell proliferation, migration, and differentiation [39]. Collagen plays a significant role in the inflammatory phase of wound healing as it activates the clotting cascade when exposed due to an injury. A study showed that the use of a collagen matrix accelerated the inflammatory response and resolved faster, thereby speeding up the wound healing process [66]. Collagen is also important in angiogenesis, as ECM remodeling provides structure for vascularization, and collagen is one of the most abundant proteins in the ECM. Depending on the type, collagen can either promote or inhibit angiogenesis. Specifically, the C-propeptide fragment of collagen I aids in recruiting endothelial cells, which are vital for blood vessel development [67]. Additionally, collagen is heavily involved in ECM reconstruction, which is a key component of normal wound healing. However, abnormal wound healing is often associated with excessive collagen deposition and irregular collagen fibre orientation. Thus, both the deposition and orientation of collagen fibres are critical factors in wound healing and ECM remodeling.

M. Albanna et al. printed a fibrin–collagen hydrogel matrix in situ for wound healing in mice and pigs. The printing process was conducted in layers, starting with the deposition of acellular hydrogel, followed by the printing of the hydrogel seeded with fibroblasts, and finally, the printing of the hydrogel containing keratinocytes. Each layer was crosslinked with thrombin prior to printing of the subsequent hydrogel layer [68]. In another study, A. Levin et al. collaborated with KIKA to modify their commercial robotic arm by incorporating sensors for detecting real-time movement, such as the animal’s breathing. They printed Viscoll, a porcine–collagen hydrogel, on rats and mini pigs. It was found that the collagen resulted in accelerated wound healing and was superior compared to using a handheld printer [69]. Similarly, L. Pontiggia et al. printed human dermo–epidermal skin substitutes (DESS) composed of collagen type I hydrogel using a robotic system. The robotic system printed the epidermis and dermis using collagen hydrogel with keratinocytes, fibroblasts, melanocytes, and human dermal microvascular endothelial cells (HDMECs). The printed DESS was transplanted onto mice, where it was found to accelerate wound healing as well as induced vascularization and pigmentation [70]. In another study, D. Campos et al. used an in situ bioprinter to print cell-laden collagen-agarose-based hydrogel containing human dental pulp cells (hDPCs) and HUVECs directly into human and bovine teeth for pulp regeneration [71].

#### 3.2.3. Alginate and Sodium Alginate

Alginate is another natural material used in many biomedical applications. It is a polysaccharide found in brown algae or Phaeophyceae [39]. Its molecular weight is 194.14 g/mol and its structure is linear [72]. Sodium alginate (SA) is the salt form of alginate, where sodium ions are bound [73]. SA is water-soluble, hydrophilic, shear-thinning, biocompatible, and inexpensive, making this desirable for biomedical applications [6,74]. Its rheological properties can also be fine-tuned through pre-crosslinking and adding thickening agents to improve its printability. Alginate has been used in wound healing due to its high biocompatibility, ability to form gels, and high absorption capacity, making it ideal for absorbing wound exudate. It also has hemostatic properties that help stop bleeding, accelerating the blood clotting and inflammatory stages of wound healing. Additionally, alginate provides a protective barrier, minimizing the risk of bacterial infections. Overall, it supports the wound healing process, particularly in bleeding wounds. However, it is not ideal for encapsulating cells, as it results in poor cell viability due to the absence of cell-binding moieties, such as RGD sequences [39]. Therefore, SA is a strong candidate for applications where cell adhesion is not required. In addition, it has a lower viscosity and mild gelation, resulting in a low mechanical strength, limiting its ability to print large structures [39]. The most common crosslinking method for alginate is ionic crosslinking using calcium chloride, as this has been shown to have higher cytocompatibility, however, calcium sulfate can also be used, as well as covalent and thermal crosslinking [39,52,75,76]. An interesting study conducted by L. Li et al. involves the use of an in situ portable skin pen to print alginate bioink containing M2 macrophages-derived extracellular vesicles (EVs). The EVs are embedded in sodium alginate bioink and crosslinked with calcium carbonate [77]. In another study, X. Wang et al. used microalgae in alginate/GelMA hollow fibre scaffold for in situ printing on mice. They evaluated the effects of oxygen production from the scaffold, induced by light exposure, on wound healing. Calcium chloride was used along with UV light for crosslinking alginate and GelMA [78].

#### 3.2.4. Chitosan

Chitosan is deacylated chitin, which is naturally found in the exoskeleton of crustacea, insects, algae, and the cell wall of fungi [79,80]. It has a large range of molecular weights which can be categorized into three groups: low molecular weight with less than 150 kDa, medium molecular weight between 150 and 700 kDa, and high molecular weight which is greater than 700 kDa [81]. It is hydrophilic, shear-thinning, and is a linear polysaccharide with a semicrystalline structure which is insoluble in neutral and basic conditions. Therefore, chitosan requires an acidic solution with pH less than 5 to be fully soluble, which allows for easy pH crosslinking [39]. This characteristic of chitosan poses challenges for its applications, particularly in bioprinting cell-laden scaffolds, as an acidic environment is toxic for cells [81,82]. The bioprinting process can be performed by extruding chitosan in acidic conditions, followed by quickly neutralizing the bioink to polymerize it. Sodium hydroxide and acetic acid are commonly used for bioprinting chitosan, however, it was found that high concentrations of sodium hydroxide results in faster gelation but poor attachment between the printed layers. Therefore, it is important to determine the optimal concentration for crosslinking [39]. Furthermore, chitosan can become thermosensitive and be thermally crosslinked when mixed with polyol-phosphate salts, such as β-glycerophosphate (BGP). BGP is commonly used with chitosan to thermally crosslink at physiological temperature of 37 °C, making it ideal as an injectable hydrogel. In addition, it has been shown that a chitosan and BGP mixture will remain a liquid at room temperature, even when the pH is above 6.3 [83,84]. Chitosan has recently gained increasing interest due to its properties, including anti-inflammatory, antitumor, antifungal, and bacteriostatic activities [83]. It has been reported that chitosan with molecular weights above 9.3 kDa and 83% degree of acetylation will result in an antibacterial property [83]. Additionally, chitosan is biodegradable through lysozymes, gastrointestinal enzymes, and colon bacteria, making it well-suited for clinical applications [84].

#### 3.2.5. Fibrin

Fibrin is a fibrillar protein formed when thrombin cleavesfibrinogen through the blood coagulation pathway. It has a molecular weight of 340 kDa, and the fibrin monomers are linear in structure [85]. Fibrin is hydrophobic, however, fibrinogen is hydrophilic [86]. As it is naturally present within the blood, it is commonly used in bioinks as it is biocompatible and biodegradable [87]. Additionally, it contains RGD sequences which act as cell adhesion sites for cells, including endothelial cells and fibroblasts [39]. ECM proteins also bind to fibrin to regulate cell processes, for example, the recruitment of monocytes or fibroblasts during granulation [88].

Fibrin gelation can be induced using thrombin, which is also part of the coagulation pathway. However, fibrin has a low mechanical strength and is therefore commonly mixed with other polymers for bioprinting [39]. In an interesting study, M. Albouy et al. used a robotic arm for in situ bioprinting, requiring 3D scanning to determine the printing path. A composite bioink derived from alginate, gelatin, and fibrinogen was printed and crosslinked with calcium chloride [89]. In another study, R. Cheng et al. investigated the use of a handheld in situ bioprinter to print cell-laden sheets containing mesenchymal stem cells (MSCs) onto inclined wound surfaces in pigs. The printer contains two separate syringes, one for the fibrin ink and one for thrombin, which crosslinked the material after mixing [90].

#### 3.2.6. Gelatin and GelMA (Gelatin Methacryloyl)

Gelatin is a hydrophilic protein derived from denatured collagen as it loses its secondary and tertiary structures [39]. Its molecular weight varies and largely depends on the extraction method and preparation techniques, however, the most common are between 100 and 300 kg/mol [52]. Gelatin is a heterogenous linear polypeptide and is shear-thinning [91,92]. Immunogenicity is not a significant concern when using gelatin as it is derived from denatured collagen, which reduces the number of immunogenic epitopes. Gelatin is commonly used as it possesses similar desirable traits as collagen, including biocompatibility, softness, fast biodegradation rate, and its porous scaffold provide structure and support for cells, such as fibroblasts, to proliferate and migrate, as well as the promotion of cell attachment, as it contains the RGD sequence [37]. Furthermore, gelatin scaffolds have high absorbance, aiding in the absorption of exudate and blood [93]. Gelatin is thermoresponsive, thus it can be crosslinked through temperature changes, with gelation typically occurring at 37 °C. However, using gelatin alone will result in reversible crosslinking, which is undesirable. Hence, it is usually combined with other polymers or is chemically modified to prevent the reversible reaction [39]. M. Zhao et al. used an in situ printing robot, requiring 3D scanning of the wound to generate the printing path for extrusion of alginate-gelatin hydrogel (AG) with platelet rich plasma (PRP) to speed up blood clotting and wound healing. Finally, calcium chloride was used for crosslinking, which took 15 min [94]. Similarly, H. Wang et al. used an in situ printer to deposit a SA-gelatin ink on abnormal wound geometries at different depths within the skin, including muscle, dermis, and epidermis [95]. SA-gelatin was also used by H. Ding et al., who used images of the wound to generate the printing path for in situ printing on a phantom burn wound. Similarly, calcium chloride was used as the crosslinking agent [96].

A promising bioink which was chemically modified from gelatin, is gelatin methacryloyl, commonly known as GelMA. The amine groups of gelatin are modified with methacrylate groups, resulting in the formation of GelMA [39]. As GelMA is synthesized through the chemical modification of gelatin, it is a partially synthetic material. The molecular weight of GelMA is measured in bloom strength, which is based on the molecular weight of gelatin. GelMA typically has a bloom strength between 50 and 300, is hydrophilic, linear in structure, and is shear-thinning [97,98,99]. GelMA is highly promising due to its ease of synthesis, low cost, ability to fine-tune physical properties, great biocompatibility, and is easy to crosslink [100]. Due to methacrylation, GelMA can be photocrosslinked, which is advantageous as it enables rapid crosslinking using light, allowing for the printing of complex structures and multiple layers. However, the drawback of photocrosslinking requires a photoinitiator, which can be slightly toxic, however, studies show it does not significantly impact cell viability if used in low concentrations [11]. The type of light can also affect its biocompatibility, as UV light, while the fastest, is more toxic compared to blue or visible light. In a study, H. Chen et al. used a robotic in situ printer to print GelMA hydrogel containing mice epithelial stem cells and skin-derived precursors (SKPs) on mice to evaluate wound healing and hair follicle regeneration. GelMA was mixed with LAP, and UV light of 405 nm was used for rapid crosslink [101]. In another study, F. Zhou et al. also used GelMA embedded with copper-containing bioactive glass nanoparticles (Cu-BGn) for printing on mice via a handheld “SkinPen”. US and UV radiation were used sequentially to crosslink the ink [32]. Similarly, J. Lee et al. also used an in situ bioprinter with optic fibre in the nozzle to print GelMA for muscle tissue restoration in mice. The optic fibre allows for UV radiation to pass through and crosslink the GelMA as it is being extruded, facilitating rapid crosslinking as it exits the nozzle [102].

#### 3.2.7. dECM

The extracellular matrix (ECM) is a large network of proteins and other molecules, providing support and nutrition to surrounding cellular components within the tissues. The ECM consists of numerous proteins, mainly including collagen and elastin, but also laminin, fibronectin, vitronectin, and tenascin, as well as proteoglycans and glycosaminoglycans (GAGs). The ECM aids in cell attachment and communication with the adjacent cells, playing imperative roles in cell growth, cell movement, and tissue maturation. Providing the essential proteins for ECM remodeling is crucial, as it is the final step in wound healing and restores the skin’s tissue architecture.

The decellularized skin extracellular matrix (dECM) is healthy skin which has gone through decellularization, a process which removes cellular components to minimize the undesired immunogenicity while preserving crucial proteins found within the native skin. There are various types of decellularization techniques including chemical, physical, and enzymatic [11]. This process is unique as it removes cells and DNA content while maintaining important proteins and the natural ECM structure, preserving both the mechanical and chemical properties of the skin [11]. Its contact angle is approximately 70 degrees, which is slightly hydrophilic [11]. It was found that when ECM is derived from the same tissue type, cell viability is improved. Additionally, maintaining the physical ECM structure helps modulate cellular activities including cell growth, proliferation, adhesion, and migration [103]. ECM has desirable biodegradability and biocompatibility, however, it undergoes thermal crosslinking, which is a slower and milder process. Consequently, it is challenging to print complex structures as the ink does not crosslink quickly enough and has low mechanical strength. Therefore, dECM is commonly blended with other materials to improve crosslinking and provide structural support.

**Figure 4:**
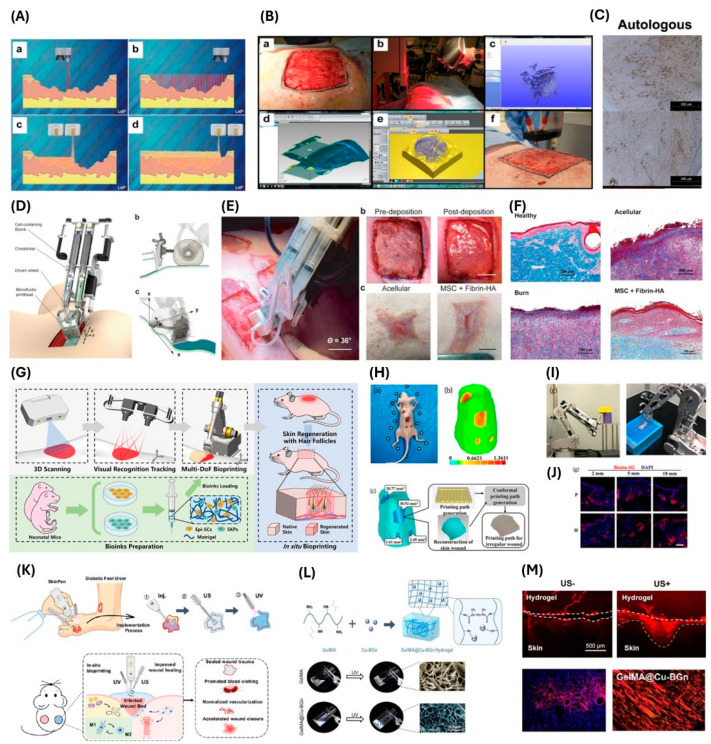
In situ bioprinting for skin applications. (**A**) Schematic illustrating the skin bioprinting concept involving 3D scanning of wound bed to capture wound topography followed by robotic bioprinting for wound coverage [68]. (**B**) The bioprinting process on a porcine wound model. A full-thickness wound (**top left**) was 3D scanned (**top middle**) to create a 3D model of the wound bed for generating the printable 3D model and slicing codes (**top right**, **bottom left** and **bottom middle**), followed by in situ robotic bioprinting on the wound (**bottom right**) [68]. (**C**) CD31-positive blood vessels of autologous cell-treated wound at week 4 (**top**) and week 8 (**bottom**) [68]. (**D**) Schematic of a handheld bioprinter consisting of two syringes for fibrinogen and thrombin, as well as a wheel to allow for easy maneuver and stable deposition of biomaterial [90]. (**E**) The use of handheld bioprinter on an inclined full-thickness porcine wound at 36 degrees (**left**). The pre- and post-deposition of biomaterial via the handheld printer on the porcine wound (**top**) and gross images of the wounds treated with acellular bioink or with MSCs encapsulated bioink after 28 days [90]. (**F**) Masson’s Trichrome stain in healthy, acellular, burn alone, and MSC plus fibrin treated groups [90]. (**G**) Schematic illustrating the in situ robotic bioprinting process including 3D scanning, visual recognition tracking, and multi-DOF bioprinting with matrigel encapsulated with Epi-SCs and SKPs for hair-follicle-inclusive skin repair [104]. (**H**) Gross image (**top left**) and thermal image (**top right**) of the murine wound model, along with the 3D model and the calculated printing path for bioprinting (**bottom**) [104]. (**I**) Robotic arm for in situ bioprinting on the back of the mouse [104]. (**J**) Staining of sebaceous glands (SG) through IF staining of biotin in bioprinted group (P) and hand implantation group (H) [104]. (**K**) Schematic illustrating the use of the SkinPen for diabetic foot ulcer through extruding the biomaterial, followed by US and UV crosslinking [32]. (**L**) The biomaterial consists of GelMA encapsulated with copper-containing bioactive glass nanoparticles (Cu-BGns) (**top**), and after UV crosslinking they resulted in a gelled porous structure (**bottom**) [32]. (**M**) Fluorescent images of the interface between porcine skin and hydrogel with or without US treatment (**top**). Fluorescent images showing CD31 stain (red) of wound treated with GelMA@Cu-BGn (**bottom left**) and Sirius red staining of the wound treated with GelMA@Cu-BGn on day 14 (**bottom right**) [32]. The permission to use the figures from Cheng et al., 2018 was obtained from Copyright Clearance Center, Inc. (Danvers, MA, USA).

In conclusion, both synthetic and natural materials have their advantages and drawbacks. Synthetic materials are ideal for controlling physical properties as their characteristics can easily be manipulated during synthesis. They also have consistent properties between varying batches. However, they lack proteins and growth factors found within the native skin which would accelerate tissue regeneration. Natural materials contain the important structure and chemical properties of the natural skin, although their physical properties are difficult to control and vary between batches. Hence, it is common to combine both materials as they can yield the desired characteristics that each material lacks individually. Figure 4 highlights novel in situ bioprinting applications of these materials and their various crosslinking mechanisms.

## 4. In Situ Deposition of Hydrogel

**Figure 5:**
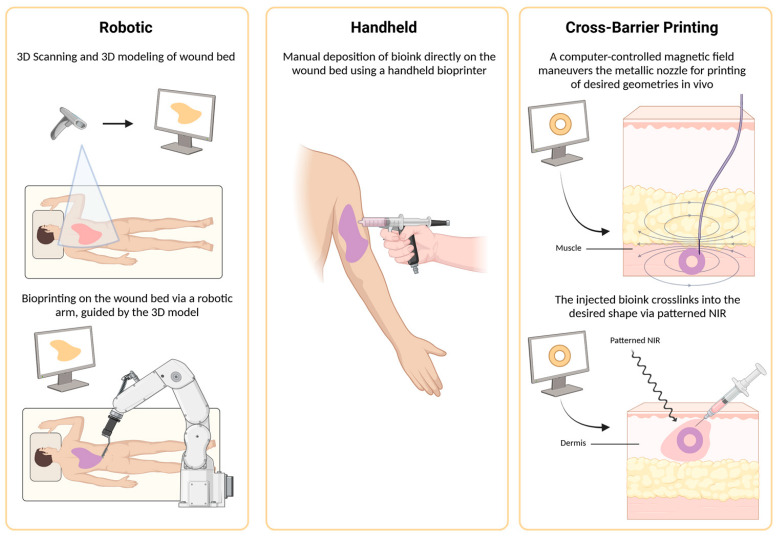
A schematic representing the three types of in situ bioprinting: robotic, handheld, and cross-barrier printing. Robotic bioprinting involves 3D scanning and 3D modeling of the wound bed to determine the printing path of the robotic arm for bioprinting on the wound. The handheld bioprinter is controlled and operated by the user to deposit bioink directly onto the wound bed. Lastly, cross barrier printing can be conducted using magnetic field and NIR. Computer-controlled magnetic field maneuvers the metallic nozzle in vivo to bioprint the desired shape as determined by the 3D model. Similarly, a patterned NIR shaped by the 3D model crosslinks the bioink injected under the skin into the desired shape. Created in BioRender. Jeschke, M. (2025) https://BioRender.com/f61p551 (accessed on 7 January 2025).

There are three primary types of bioprinting: extrusion, inkjet and laser-assisted bioprinting (LAB) [105]. Each method has its own advantages and drawbacks that must be carefully considered when selecting the appropriate technique for a specific application. Additionally, there are various delivery mechanisms available, including handheld, robotic, and cross-barrier printing (Figure 5). The optimal printing parameters reported in the literature for various printing types are also discussed, along with a literature review on the use of in situ bioprinting for skin regeneration over the past six years (Table 1).

### 4.1. Extrusion, Inkjet, LAB

Extrusion-based printing is the most common method. Typically, pneumatic pressure is the mechanism used for bioink extrusion, however a piston or screw may also be used [105]. The bioink is deposited as a continuous filament and depending on the crosslinking mechanism and mechanical strength of the ink, it can be printed in layers. Extrusion is particularly favoured for handheld printers, especially for in situ printing applications that require rapid crosslinking. This method is compatible with skin applications, as this can print substances with higher elastic variation and accommodate higher cell densities encapsulated within the ink [105].

Inkjet printing is the second most common and is based on droplet deposition. Inkjet printing can be categorized into three types: continuous-inkjet, electro-hydrodynamic jet, and drop-on-demand inkjet, with the latter being the most common [106]. Drop-on-demand inkjet printers include thermal, piezoelectric, and electrostatic printers. These printers use heat or electricity to vaporize bubbles, thereby ejecting ink droplets from the nozzle [106]. While inkjet printers allow for rapid printing, they are less common due to their inability to print a continuous flow and require lower cell densities. Due to their properties, inkjet printers are not suitable for in situ applications.

Lastly, LAB is the least common method due to its speed limitations. This method contains a ribbon made of transparent glass, laser absorbing metal, and a suspended layer of bioink [107]. Laser pulses are directed at the ribbon, causing vaporization that forms high-pressure bubbles, which pushes bioink droplets to fall onto the receiving substrate [107]. LAB offers significant advantages, including extremely high precision, resolution, and can print high cell densities (~10^8^ cells/mL) [107]. However, this method is time consuming and is not applicable for printing large geometries, which limits its application in situ.

### 4.2. Handheld Printers

Handheld printers are compact devices that can be held by the user, providing full control of the printing process. A typical design is similar to a pen or a gun, where the user holds a button to initiate ink deposition and releases to stop printing. These devices are favourable due to their user-friendly design, the ability for users to control the printing process, and some printers can print sheets instead of filaments, providing faster wound coverage. However, they have a lower resolution and are limited in their capacity to print large volumes due to their restricted storage of bioink. Hence, handheld printers would be ideal for rapid printing to provide wound coverage on smaller, flat wounds such as on the back or abdomen. F. Zhou et al. used a handheld “SkinPen” to print GelMA and Cu-BGn bioink in situ on STZ-induced diabetic rats infected with *S. aureus*, followed by sequential crosslinking using US and UV radiation. The treated wounds did not show signs of inflammation due to the excellent antibacterial properties of Cu-BGn, and CD206 expression was significantly higher than the control, indicating an increase in M2 macrophages and improved wound healing [32]. In another study, N. Hakimi et al. developed a portable printer designed to print small alginate-fibrin sheets. This printer features numerous nozzles, allowing for the deposition of small sheets, and incorporates a wheel to aid in the printing process. The sheets undergo mild crosslinking using thrombin and calcium chloride, and the ink encapsulated fibroblasts and keratinocytes. The printer was tested on mice and pigs to evaluate its printability and cytotoxicity, however, its wound healing was not measured in detail [60]. In addition, R. Cheng et al. used the same handheld printer on full-thickness wounds in pigs, where fibrinogen sheets containing MSCs were crosslinked using thrombin. Wound healing was assessed in depth through immunofluorescent staining for biomarkers associated with angiogenesis, macrophages, and proliferation, as well as evaluating scars using the Vancouver Scar Scale. The results indicated that the treated wounds did not show a significant re-epithelialization rate compared to the acellular ink, but had significantly better scarring, blood vessel formation, and M2 macrophage presence [90]. Interestingly, L. Li et al. used an in situ portable skin pen to print alginate ink containing M2 macrophage-derived extracellular vesicles (EVs) on BALB/c mice. The treated wounds exhibited denser microvessels, thicker collagen deposition, and a smaller wound size [77]. In another study, W. Li et al. developed a novel application of using a smartphone for in situ printing, where a smartphone connects to a projector to provide the 3D model for printing through an application installed on the device. The projector projects the image through various lenses for in situ printing. PEGDA, GelMA, and allylated gelatin (GelAGE) encapsulated C2C12 cells and was crosslinked using visible light with Ru/SPS photoinitiator [108].

### 4.3. Robotic Printers

Robotic printers are another type of in situ bioprinting. Robotic arms are the most common design which are programmed to detect wound morphology, calculate the printing path, and print within the wound area. These robotic printers must remain fixed in place to accurately determine the coordinates for the printing path. Their advantages include the ability to print complex structures at higher resolutions, tailored to the wound shape as determined by built-in 3D scanners, commonly integrated into robotic printers. Additionally, they can print large volumes due to their significant storage capacity. However, the drawbacks include the requirement for user training as the operation can be complex, and a slower printing speed as it only prints filaments. Thus, robotic printing would be more suitable for printing larger, complex structures that require high resolution and have less time constraints. For instance, this would be applicable for complex wound shapes which require a better fit for improved scarring, as differences in resolution have been shown to impact wound healing outcomes [69].

W. Zhao et al. used a robotic arm equipped with a 3D scanner and binoculars to scan the wound and calculate the printing path. The robotic arm printed Matrigel bioink containing epidermal stem cells (Epi-SC) and SKPs in mice, using thermal crosslinking facilitated by the body temperature of the mouse [104]. Similarly, M. Albouy et al. also used a robotic arm with a built-in 3D scanner to print alginate, gelatin, and fibrinogen ink on porcine wounds. Cell-laden bioink with encapsulated dermal fibroblasts demonstrated superior wound healing effects compared to the acellular bioink [89]. In another study, H. Chen et al. printed GelMA, containing Epi-SCs and SKPs, on BALB/c nu/nu mice via a robotic arm. LAP was used as the photoinitiator, and the gel was crosslinked using 405 nm UV light. The results indicated that the treated wounds healed successfully, containing hair follicles, sebaceous glands, epidermis, dermis, and blood vessels [101]. Furthermore, A. Levin et al. collaborated with KIKA to modify their robotic arm for in situ printing. This arm features sensors allowing for real-time sensing and adjustment in response to movements such as the animal’s breathing. It contains a 3D scanner to define the printing path and deposited Viscoll, a porcine collagen hydrogel, containing platelet lysate, human fibroblasts (HF), and human umbilical vein endothelial cells (HUVECs) in Wistar rats and Wiesenau pigs. The performance of the robotic arm was compared to that of a handheld printer, showing that the robotic arm resulted in superior wound healing outcomes due to its higher printing resolution [69].

### 4.4. Cross-Barrier Printing

Cross-barrier printing is a minimally invasive method of bioprinting, and is particularly advantageous, allowing for deposition of bioinks in vivo without the need for creating large incisions. In a study conducted by Y. Chen et al., non-invasive bioprinting was achieved through the injection of GelMA in mice. The ink was polymerized in vivo using patterned NIR light, which was modelled using AutoCAD. The patterned NIR crosslinked the GelMA beneath the skin into the modelled shape [109]. In another study, C. Zhou et al. used a ferromagnetic soft catheter composed of PDMS and NdFeb to deposit bioink in vivo onto various organs. The catheter was inserted through a small incision in mice, and its movement was manipulated by a magnetic field controlled through the computer [110]. Both studies used innovative approaches for delivering bioinks in the desired shape while minimizing the need for large surgical interventions. Although the area of minimally invasive printing remains unexplored, it has been gaining increasing attention.

**Table 1:** Literature review on the use of in situ bioprinting for skin regeneration and an assessment of their wound healing outcome. To facilitate the assessment of bioprinting and wound healing efficacy, a scale was used to evaluate seven wound healing parameters: wound closure, epithelialization speed, contraction, inflammation, blood vessel formation, M1 to M2 macrophage shift, and scarring. The following scale was established: (1) no significant difference compared to the control, or the outcome was not mentioned, (2) significantly better than the control, and (3) significantly better than the control and other treatment groups. The wound healing efficacy is the sum of the individual wound healing property scores, with a higher score indicating better wound healing outcomes.

In Vivo Assessment of In Situ Bioprinting and Wound Healing Outcomes								
Year	Author	Printing Type	Biomaterials	Crosslink Mechanism	Encapsulated Cells	Animal Model	Wound Healing Properties	Wound Healing Efficacy
2019	M. Albanna [68]	Robotic	Fibrin/collagen	Thrombin	Fibroblasts/ Keratinocytes	Nu/nu mice, SPF Yorkshire pigs	Wound closure = 3 Epithelialization = 3 Contraction = 1 Inflammation = 2 Vessel = 1 Macrophage = 1 Scar = 1	12
2019	N. Hakimi [60]	Handheld	Alginate/fibrin/ collagen/HA	CaCl2, Thrombin	Fibroblasts/ Keratinocytes	Murine and Porcine	Wound closure = 1 Epithelialization = 1 Contraction = 1 Inflammation = 2 Vessel = 1 Macrophage = 1 Scar = 1	8
2020	R. Cheng [90]	Handheld	Fibrinogen	Thrombin	MSCs	Porcine	Wound closure = 1 Epithelialization = 2 Contraction = 3 Inflammation = 2 Vessel = 2 Macrophage = 3 Scar = 3	16
2021	C. Zhou [110]	Robotic	Ecoflex/PDMS-1700, silver ink, hydrogel ink	None	None	Sprague Dawley Rats	Tested printability in rats but did not assess wound healing outcomes	-
2022	M. Albouy [89]	Robotic	Alginate/gelatin/ fibrinogen	CaCl2, Thrombin	Fibroblasts	Porcine	Wound closure = 1 Epithelialization = 1 Contraction = 1 Inflammation = 1 Vessel = 1 Macrophage = 1 Scar = 2	8
2022	X. Wang [78]	Robotic	Microalgae Alginate/GelMA	CaCl2 and UV (365 nm)	Chlorella Pyrenoidosa (microalgae cells)	C57BL/6 mice	Wound closure = 2 Epithelialization = 2 Contraction = 1 Inflammation = 1 Vessel = 3 Macrophage = 1 Scar = 1	11
2022	M. Zhao [94]	Robotic	Alginate/Gelatin with Platelet Rich Plasma	CaCl2	Fibroblasts and Epidermal Stem Cells	Sprague-Dawley Rats	Wound closure = 3 Epithelialization = 1 Contraction = 1 Inflammation = 3 Vessel = 3 Macrophage = 3 Scar = 1	15
2022	W. Zhao [104]	Robotic	Matrigel	Thermal	Epidermal Stem Cells and Skin-Derived Precursor Cells	C57BL/B6 and BALB/c nu/nu mice	Wound closure = 1 Epithelialization = 1 Contraction = 1 Inflammation = 1 Vessel = 1 Macrophage = 1 Scar = 1	7
2023	H. Chen [101]	Robotic	GelMA	UV (405 nm)	Epidermal Stem Cells and Skin-Derived Precursor Cells	BALB/c nu/nu mice	Wound closure = 1 Epithelialization = 1 Contraction = 1 Inflammation = 1 Vessel = 1 Macrophage = 1 Scar = 1	7
2023	A. Levin [69]	Robotic	Viscoll/ platelet lysate	Thermal	Fibroblasts, HUVECs	Wistar rats and Wiesenau minipigs	Wound closure = 1 Epithelialization = 1 Contraction = 1 Inflammation = 2 Vessel = 2 Macrophage = 1 Scar = 1	9
2023	L. Li [77]	Handheld	Sodium alginate	CaCO3	M2-derived EVs	Balb/C mice	Wound closure = 2 Epithelialization = 1 Contraction = 1 Inflammation = 1 Vessel = 2 Macrophage = 2 Scar = 1	10
2023	F. Zhou [32]	Handheld	GelMA with Cu-BGn	US and UV (365 nm)	None	STZ-induced diabetic rats	Wound closure = 3 Epithelialization = 1 Contraction = 1 Inflammation = 2 Vessel = 3 Macrophage = 3 Scar = 1	14
**In Vitro Assessment of In Situ Bioprinting**								
**Year**	**Author**	**Printing Type**	**Biomaterials**	**Crosslink** **Mechanism**	**Encapsulated Cells**	**Animal Model**	**Wound Healing Properties**	**Wound Healing Efficacy**
2018	H. Ding [96]	Robotic	Gelatin/Sodium Alginate	CaCl2	Fibroblasts	None	-	-
2020	H. Wang [95]	Robotic	Gelatin/Sodium Alginate	-	None	None	-	-
2021	W. Li [108]	Robotic	PEGDA, GelMA, GelAGE	Visible light	C2C12	None	-	-
2021	Y. Tianyuan [43]	Handheld	PEGDA, gelatin/sodium alginate, PCL	UV (365 nm)	None	None	-	-
2023	E. Pagan [51]	Handheld	PEGDA-Laponite, PLGA, PNIPAM, alginate, GelMA	UV (405 nm), CaCl2	Fibroblasts, HaCaT, C2C12, SKOV-3	None	-	-

### 4.5. Critical Criteria and Parameters Influencing Printing Quality

Successful bioprinting involves optimizing various parameters, including printing speed, pneumatic pressure, nozzle size, and temperature. It is of utmost interest to bioprinting experts to select the optimal printing parameters tailored to the biomaterials used and cellular contents incorporated. A potential range of optimal printing conditions depending on the different printing mechanisms, introduced in the current manuscript, are summarized in Table 2 to offer insights for further optimization. However, these factors present a trade-off between cell viability and printability. Encapsulated cells typically cannot withstand harsh conditions, such as acidic environments, high or low temperatures, or high amount of shear stress generated during extrusion. Hence, achieving high printing quality while maintaining cell viability presents a significant challenge and represents a complex optimization problem.

Printing speed and pneumatic pressure are closely related parameters which significantly impact the smoothness of extruded filaments and cell viability. Excessively high printing speeds and pressures can disrupt the filaments during deposition, and too slow of a printing speed and low pressure can result in the formation of significantly thicker filaments, resulting in low resolution. Furthermore, cell viability decreases with increasing pneumatic pressure as the associated shear stress can be harmful to the cells. Therefore, it is crucial to determine the optimal combination of speed and pressure to achieve smooth filament deposition while preserving cell viability.

Nozzle size also profoundly affects printing resolution, a relationship that is intuitively understood. Large needle gauges produce larger filaments, which are ideal for printing large constructs, however this results in an extremely low resolution when printing fine details. Alternatively, smaller needle gauges allow for printing intricate details at higher resolutions, but yield extremely small filaments, which is ideal for printing small constructs. Therefore, the selection of the nozzle size must be tailored to the specific requirements of the detail level and construct size.

Temperature is closely related to several properties of bioinks including viscosity, polymerization, and cell viability. Most materials exhibit varying viscosities as a function of temperature. Generally, higher temperatures result in reduced viscosity, and lower temperatures lead to increased viscosity. These variations significantly affect printing resolution; excessively watery inks may fail to hold its shape, while extremely viscous inks are difficult to extrude. Additionally, temperature affects the polymerization of certain materials that undergo thermal crosslinking, such as collagen and dECM. These materials typically require cooler temperatures to remain a liquid, while higher temperatures facilitate crosslinking. Furthermore, temperature affects the viability of encapsulated cells. While 37 °C is ideal for mammalian cells, the required printing temperature for specific bioinks is typically much lower, which can be detrimental to cell health if maintained for a prolonged period.

Another critical parameter to be considered for in situ printing is the cell viability and cellular functions post-printing when incorporating cells within the bioink. Cells, especially stem cells, play a major role in tissue regeneration. Target cell type differentiations give rise to appropriate tissue phenotype formation, recruitment of other cells from surrounding tissues and organs, and the promotion of tissue maturation, especially aiding in anti-inflammation during tissue remodeling and tissue vascularization. With the proper bioink designed, various cell types ranging from keratinocyte, melanocyte, fibroblast, adipocyte, as well as endothelial cells, can be incorporated within the printed skin substitutes. This enables the recapitulation of the native skin and thereby accelerating the neo-tissue maturation and accelerate skin wound healing to its normal functions [111]. In addition, with the proper cell population, such as M2 macrophages at the wound site, cell-laden in situ bioprinting may further contribute to scarless healing, with the optimal inflammatory response orchestrated in favor of a less inflamed healing process during the skin regeneration.

Indeed, bioprinting is complex as it requires optimization of numerous parameters for each material, primarily focusing on the balance between printability and cell viability. In the future, it is vital to develop a mathematical model capable of approximating the optimal printing parameters for various material combinations. However, this would require an extensive design of experiment (DOE) and a comprehensive database of all possible materials and their combinations, along with the optimal parameters for each printing mechanism. Developing a mathematical model would significantly advance the area of bioprinting, ultimately saving time, effort, and costs associated with numerous experimentations required to identify optimal printing conditions.

**Table 2:** General considerations of the potential range of optimal printing parameters for various printing mechanisms, including printing speed, pressure, size or resolutions, and nozzle temperature, as reported in the literature.

	Printing Speed	Pressure	Size/Resolution	Nozzle Temperature	References
Extrusion	4–8 mm/s	100–200 kPa	30–32 G	26–29 °C	[112,113]
Inkjet	0.88–2.08 m/s	22–40 V	23 G	300 °C (thermal printers)	[114,115]
LAB	5 kHz	Laser energy: 6–16 uJ	5–18 mm	37 °C	[116,117]
Handheld: single nozzle	4–8 mm/s	100–200 kPa	30–32 G	26–29 °C	[112,113]
Handheld: sheet deposition	4–10 mm/s	Syringe pump: 150–1000 µL/min	8–20 mm width	25 °C	[60]
Robotic	4–8 mm/s	100–200 kPa	30–32 G	26–29 °C	[112,113]

## 5. Clinical Translation

### 5.1. Space and Logistical Considerations

Despite significant investment in research and the development of validation methods for in situ bioprinting of skin, several logistical and practical challenges remain that must be addressed before bioprinting can effectively be used in the operating room. One logistical concern is the space required within the operating room, which is already crowded with personnel and equipment. The size of the printing station makes it cumbersome to use in such a setting. Bioprinters, such as tabletop extrusion printers that require a dedicated desktop, or robotic printers, may not be realistic in the operating room. Conversely, handheld printers such as the SkinPen would allow the surgeon to bioprint the skin directly in situ, thereby avoiding an additional large workstation in the operating room.

### 5.2. Sterility Compliance

Similar to any tool used within the sterile surgical field, a bioprinter, whether handheld or robotic, must comply with sterility guidelines. Thus, the design requirements for handheld and robotic printers would stipulate that bioprinters be single use, as well as manufactured and packaged in a sterile fashion, or a robotic bioprinter must be capable of undergoing sterilization and cleaning. For intraoperative devices, a sterility assurance level (SAL) of one in one million is generally accepted [118]. Further, the substrate material and bioink of the bioprinter must be free from contamination for use in a sterile setting.

### 5.3. Feasibility in Skin-Related Surgical Operations

Acute and chronic wounds are prevalent concerns in primary care and surgical specialties. Primary care physicians regularly encounter venous ulcers and diabetic ulcers—of which roughly 25% of people living with diabetes will develop [119]. General surgery, orthopaedic surgery, and plastic surgery are frequently involved in the management of chronic and acute wounds, including those resulting from skin and soft tissue infections, pressure ulcers, surgical site infections, and open surgical wounds. Traditionally these wounds are managed with systemic antibiotics, dressing changes, vacuum assisted closure (VAC) dressings, debridement, and potential skin grafting or surgery for definitive closure of the wound. Depending on the extent of closure required, STSG may be performed in a procedure room or in an operating room. Skin grafting, and potentially the application of bioprinted skin, is not exclusive to plastic surgery. However, its use is less common in other specialities due to of lack of familiarity and training, as well as infrequent use. Bioprinted skin could potentially be used in the management of any wound requiring skin closure, provided that the same sterility and physiological conditions are met.

Following a burn injury, the time of surgical grafting procedures depend on the wound size, and the size of the excision depends on the availability of graft material. However, as a general guideline, no more than 40% of total body surface area is excised and grafted at one time to prevent excessive blood and fluid loss [120]. Typically, traditional split thickness skin grafts require from one to four hours to complete. However, there is a lack of concrete studies in both animal and human models to compare the surgical time of bioprinted skin versus traditional skin grafting. However, it is conceivable to anticipate, that particularly in larger graphs, bioprinted skin would take longer to complete. In an acute burn surgery, increased surgery duration is associated with an increased length of stay in hospital [121]. However, the same study found that the use of autologous skin cell spray reduced the surgical time as well as the length of hospitalization. In general, prolonged surgical time is associated with an increased risk of wound dehiscence, fluid loss, infection, graft failure, and deep vein thrombosis [122]. Thus, reducing the operating time is another important factor to consider for improving burn care, which could potentially be improved by using bioprinted skin. Furthermore, the mean index hospitalization costs for burn patients in 2021 was $32,360 CAD, with a mean length of stay of 14.3 days [123]. Given that the length of hospitalization contributes to health care expenditure, it is crucial to optimize the length of stay for best patient outcomes while also controlling healthcare costs.

### 5.4. Training

The integration of any new device into a surgical workflow requires that all personnel involved with handling and using the device receive appropriate training. The life cycle of a surgical device involves handling and cleaning at medical device reprocessing centres, nurses preparing and setting up the device in the operating room, and surgeons using the device during surgery. Thus, throughout this life cycle, all individuals must be properly trained to use, prepare, and clean the device.

## 6. Conclusions

As an emerging and innovative tissue engineering strategy, the in situ bioprinting technique offers additional additive biomanufacturing approaches directly upon the wound area for rapid wound coverage and demonstrates potential in creating personalized geometries tailored to the complicated surface features at the wound site. Ideal biomaterials for in situ bioprinting require tunable biological and biophysical properties designed for desirable rapid material deposition and crosslinking, highly controllable printability, wide compatibility with diverse cell types, and capability of supporting cell adherence, proliferation, and differentiation. The current restrictions of the in situ bioprinting mainly lie in the suitable bioink and printing mechanism combinations to either allow multi-material dispensing or to produce cell-free or cell-laden constructs in a timely manner in clinical settings. However, such challenges do not hinder the great potential of in situ bioprinting as an appealing skin regeneration strategy, which allows high flexibility for physicians to implement rapid wound coverage in operations.
